# Benefits of Dominance over Additive Models for the Estimation of Average Effects in the Presence of Dominance

**DOI:** 10.1534/g3.117.300113

**Published:** 2017-08-25

**Authors:** Pascal Duenk, Mario P. L. Calus, Yvonne C. J. Wientjes, Piter Bijma

**Affiliations:** Animal Breeding and Genomics Group, Wageningen University & Research, 6700 AH Wageningen, The Netherlands

**Keywords:** dominance, Hardy-Weinberg equilibrium, average effect, root mean squared error, accuracy

## Abstract

In quantitative genetics, the average effect at a single locus can be estimated by an additive (A) model, or an additive plus dominance (AD) model. In the presence of dominance, the AD-model is expected to be more accurate, because the A-model falsely assumes that residuals are independent and identically distributed. Our objective was to investigate the accuracy of an estimated average effect ($\hat{\alpha }$) in the presence of dominance, using either a single locus A-model or AD-model. Estimation was based on a finite sample from a large population in Hardy-Weinberg equilibrium (HWE), and the root mean squared error of $\hat{\alpha }$ was calculated for several broad-sense heritabilities, sample sizes, and sizes of the dominance effect. Results show that with the A-model, both sampling deviations of genotype frequencies from HWE frequencies and sampling deviations of allele frequencies contributed to the error. With the AD-model, only sampling deviations of allele frequencies contributed to the error, provided that all three genotype classes were sampled. In the presence of dominance, the root mean squared error of $\hat{\alpha }$ with the AD-model was always smaller than with the A-model, even when the heritability was less than one. Remarkably, in the absence of dominance, there was no disadvantage of fitting dominance. In conclusion, the AD-model yields more accurate estimates of average effects from a finite sample, because it is more robust against sampling deviations from HWE frequencies than the A-model. Genetic models that include dominance, therefore, yield higher accuracies of estimated average effects than purely additive models when dominance is present.

In quantitative genetics, dominance is the phenomenon where the genotypic value of the heterozygote deviates from the mean genotypic value of the two homozygotes (Falconer and Mackay 1996). Dominance has been shown to play an important role in production traits of livestock species (Morris and Binet 1966; Sellier 1976; Visscher *et al.* 2000) and plant crops (Xiao *et al.* 1995; Stuber 2010; Huang *et al.* 2016). In livestock genetic improvement, however, research has been focused on the estimation of average effects, because average effects capture all heritable variation (Lynch and Walsh 1998). The average effect of a single gene (α), also known as the allele substitution effect, is defined as the linear regression coefficient of genotypic values on allele counts (Falconer and Mackay 1996). Under Hardy-Weinberg equilibrium (HWE), the α at a biallelic locus is a function of the additive (a) and dominance (d) parts of gene effects, and the population allele frequency p:

α=a+(1−2p)d, (1)

where a is half the difference in genotypic value between both homozygotes, and d is the difference between the genotypic value of the heterozygote and the average genotypic value of both homozygotes. With genomic data, additive (A) models estimate α by linear regression of phenotypes on allele counts (*i.e.*, genotypes). Additive plus dominance (AD) models estimate the additive and dominant gene effects separately, after which $\hat{\alpha }$ can be obtained from Equation 1 (Lynch and Walsh 1998). For both models, the part of dominance that is not captured by the average effect is called the dominance deviation (Falconer and Mackay 1996).

When the A-model is used, dominance deviations are not modeled and thus become part of the residual. As a consequence, the residuals are not independent and identically distributed (IID), because dominance deviations are different across genotypes (Ott and Longnecker 2010). The A-model may therefore give inaccurate estimates of α, because it falsely assumes that the residuals are IID. When the AD-model is used, dominance deviations are explicitly modeled, and the residuals will more likely be IID. In the presence of dominance, the AD-model may therefore yield more accurate estimates of α than the A-model. In contrast to the A-model, however, the AD-model requires the estimation of two effects instead of one (for a single locus), which may reduce the accuracy with which these effects are estimated. Additionally, dominance effects are generally smaller and therefore harder to estimate than additive effects (Lynch and Walsh 1998). For these reasons, the AD-model may require more individuals to be sampled for an accurate estimation of α, compared with the A-model. Furthermore, estimating dominance effects when there is very little or no dominance may lead to overfitting (Ott and Longnecker 2010). Hence, while the AD-model may better fit the data in the presence of dominance, the A-model may be preferred when the sample size is relatively small and dominance is negligible. It is, however, not yet clear how sample size and dominance effect size affect the accuracy of $\hat{\alpha }$ with the A-model *vs.* the AD-model.

The objective of this work, therefore, was to investigate the root mean squared error (RMSE) of the estimated $\hat{\alpha }$ at a single locus in the presence or absence of dominance, using either an A-model or an AD-model. We start with some theory of a single locus model, then derive the expected estimate of α, and calculate the RMSE of $\hat{\alpha }$ for several broad-sense heritabilities, dominance effects, sample sizes, and allele frequencies. We then calculate the mean RMSE for several degrees of dominance over the distribution of allele frequency, and identify mechanisms that underlie the differences between the A-model and AD-model.

## Theory

Our interest is to estimate the average effect (α) at a single locus in a large population that is in HWE, from data collected as a finite sample of that population. The average effect will be treated as a fixed effect (as in quantitative genetics), and not a random variable (*e.g.*, as in genomic prediction) (de los Campos *et al.* 2015). In quantitative genetics, α at a single locus can be estimated from the sample by linear regression using an A-model or an AD-model. The A-model estimates α directly through linear regression of phenotypic values on allele counts,

y=xα+e, (2)

where y is a vector of centered phenotypes, e is a vector of residuals, and x is a vector of centered allele counts with $\left(0-2p_{s}\right)$ for individuals with 0 copies of the alternative allele, $\left(1-2p_{s}\right)$ for individuals with one copy, and $\left(2-2p_{s}\right)$ for individuals with two copies. The term $p_{s}$ is the allele frequency of the alternative allele, observed in the sample. Throughout this paper, we will use the term genotypes to indicate the three allele count classes, with values of 0, 1, or 2. With the A-model, the ordinary least squares estimate (LSE) of α is

$${\hat{\alpha }}_{A}={\left[x^{\prime }x\right]}^{-1}\left[x^{\prime }y\right].$$ (3)

The AD-model estimates the additive (a) and dominant (d) gene effects by multiple linear regression

y=xa+md+ε, (4)

where m is a dominance indicator vector with $\left(0-2p_{s}\left(1-p_{s}\right)\right)$ for homozygous individuals, and $\left(1-2p_{s}\left(1-p_{s}\right)\right)$ for heterozygous individuals. Vectors y and x are the same as in the A-model, and ε is a vector of residuals. Note that this is the genotypic parameterization as described by Vitezica *et al.* (2013). With the AD-model, the LSE of a and d are

$$\left[\begin{array}{c} \hat{a}\\ \hat{d} \end{array}\right]={\left[\begin{array}{cc} x\prime x & x\prime m\\ m\prime x & m\prime m \end{array}\right]}^{-1}\left[\begin{array}{c} x\prime y\\ x\prime m \end{array}\right].$$ (5)

The $\hat{\alpha }$ from the AD-model is subsequently calculated as

$${\hat{\alpha }}_{AD}=\hat{a}+\left(1-2p_{s}\right)\hat{d}.$$ (6)

By definition, $\hat{\alpha }$ from both models give an estimate of the average effect in the sample (Falconer and Mackay 1996). Because the size of the sample is finite, genotype and allele frequencies in the sample might deviate from the frequencies in the total population. These deviations might introduce error in the estimation of α. To investigate the effects of finite sample size in the presence of dominance, the estimates from the A-model (${\hat{\alpha }}_{A}$) and the AD-model (${\hat{\alpha }}_{AD}$) were compared by computing their RMSE for several scenarios.

### Expectation of $\hat{\alpha }$

If we take a random sample of N individuals from a large population in HWE that has allele frequency p, the expectation of $\hat{\alpha }$ can be computed using probabilities and estimates of each possible sample composition. We define c as a set of variables $\left\{n_{0},n_{1},n_{2}\right\}$ that describe unique sample compositions, where $n_{0}$ is the number of individuals with genotype 0, $n_{1}$ is the number of individuals with genotype 1, and $n_{2}$ is the number of individuals with genotype 2. The probability of sampling c is calculated from the multinomial probability function

$$P\left(c|N,p\right)=\frac{N!}{n_{0}!n_{1}!n_{2}!}g_{0}^{n_{0}}g_{1}^{n_{1}}g_{2}^{n_{2}}.$$ (7)

Conditional variables N and p are hereafter omitted to improve readability, so that P(c|N,p) is abbreviated as P(c). The quantities $g_{0},$ $g_{1}$, and $g_{2}$ are the genotype frequencies in the HWE population, and follow from the population allele frequency p ($g_{0}={\left(1-p\right)}^{2};$ $g_{1}=2p\left(1-p\right);$ $g_{2}=p^{2}$).

The expectation of $\hat{\alpha }$ is computed as the sum over all products of probabilities P(c) and corresponding estimates $\hat{\alpha }\left(c\right),$

$$E\left(\hat{\alpha }\right)=\sum_{n_{0}=0}^{N-1}\sum_{n_{1}=0}^{N-n_{0}}I\left(c\right)P\left(c\right)\hat{\alpha }\left(c\right),$$ (8)

where $\hat{\alpha }\left(c\right)\;$is the LSE of α given c. The α cannot be estimated when the sample consists of individuals that all have the same genotype, so we use I(c) as an indicator variable to exclude such samples

$$I\left(c\right)=\{\begin{array}{rr} 0, & n_{1}=N\\ 0, & n_{0}+n_{1}=0\\ 1, & all\;other. \end{array}$$ (9)

Note that samples including only genotypes 0 are excluded from Equation 8, by summing $n_{0}$ from 0 to N−1, instead of from 0 to N. After excluding samples with I(c)=0, the probabilities P(c) of the remaining samples were rescaled so that they sum to 1.

### Root mean squared error

The RMSE is defined as the root of the expected squared difference between the $\hat{\alpha }$ estimated from the sample, and the true value of α

$$RMSE\left(\hat{\alpha }\right)=\sqrt{E\left[{\left(\hat{\alpha }-\alpha \right)}^{2}\right]}=\sqrt{\sum_{n_{0}=0}^{N-1}\sum_{n_{1}=0}^{N-n_{0}}I\left(c\right)\delta \left(c\right),}$$ (10)

where δ(c) is the contribution of finite sampling deviation to the RMSE

$$\delta \left(c\right)=P\left(c\right){\left(\hat{\alpha }\left(c\right)-\alpha \right)}^{2}.$$ (11)

We define δ(c) here because we will later on focus on the contribution of a single finite sample c to the RMSE. The above expressions will be used to investigate the effect of N, $H^{2},$ p, and d on the RMSE of $\hat{\alpha }$ with the A-model and the AD-model.

## Methods

We aim to illustrate the effect of sample size (N), broad-sense heritability ($H^{2}$), allele frequency p, and dominance effect d, on RMSE of estimated average effects ($\hat{\alpha }$). As a base scenario, we chose one for both the additive and dominance effect of the gene (*e.g.*, full dominance). The expected value of $\hat{\alpha }$ was calculated for N∈{300, 500, 1000}, $H^{2}\in \left\{0.01,\;0.05,\;1\right\},$ and p=[0.001−0.999] (increments of 0.001), with the A-model (Equation 3) and AD-model (Equation 5). The variation in broad-sense heritability was achieved by adding random residuals to the phenotypes (y). In addition, we varied the dominance effect (d∈{0, 0.1, 0.2, 0.5}) for the scenario where N=500 and $H^{2}=0.05.$

The $\hat{\alpha }\left(c\right)$ from the AD-model were computed using the sample allele frequency ($p_{s}$) in Equation 6 instead of the population allele frequency (p), because the latter is usually unknown. For samples where one of the genotypes was missing, $\hat{\alpha }\left(c\right)$ with the AD-model was computed in the same way as with the A-model, because in those cases the vector of genotypes x was completely confounded with dominance vector m.

Additionally, to quantify the average accuracy of $\hat{\alpha },$ we computed the mean RMSE of $\hat{\alpha },$ assuming a distribution for the allele frequency. For this purpose, we used the RMSE as a function of p and numerically integrated over p using its expected distribution under a drift model,

$$\text{RMSE}=\int_{\frac{1}{2N_{e}}}^{1-\frac{1}{2N_{e}}}f\left(p\right)RMSE\left(p\right)dp.$$ (12)

Here, $N_{e}$ is the effective population size, f(p) is the distribution of allele frequencies when mutation is ignored, p ranges from $1/2N_{e}$ to $1-1/2N_{e}$ (Wright 1931; Goddard 2009), and

$$f\left(p\right)=\frac{k}{2p\left(1-p\right)}.$$ (13)

To ensure that ∫f(p)dp=1, k was given a value of $1/log\left(2N_{e}-1\right).$ The resulting distribution of allele frequencies is U-shaped, and a low $N_{e}$ yields a more uniform distribution than a high $N_{e}.$ We computed the mean RMSE for several $N_{e}$ (50, 100, and 200), N (200–600), and sizes of dominance effect d (0.5, 1, and 1.5). We considered sample sizes up to 600 instead of 1000 to reduce computation time. In these scenarios, both $H^{2}$ and the additive gene effect (a) were equal to one.

The data used can be regenerated exactly following the descriptions in this paper.

## Results

### Root mean squared error

**Figure 1:**
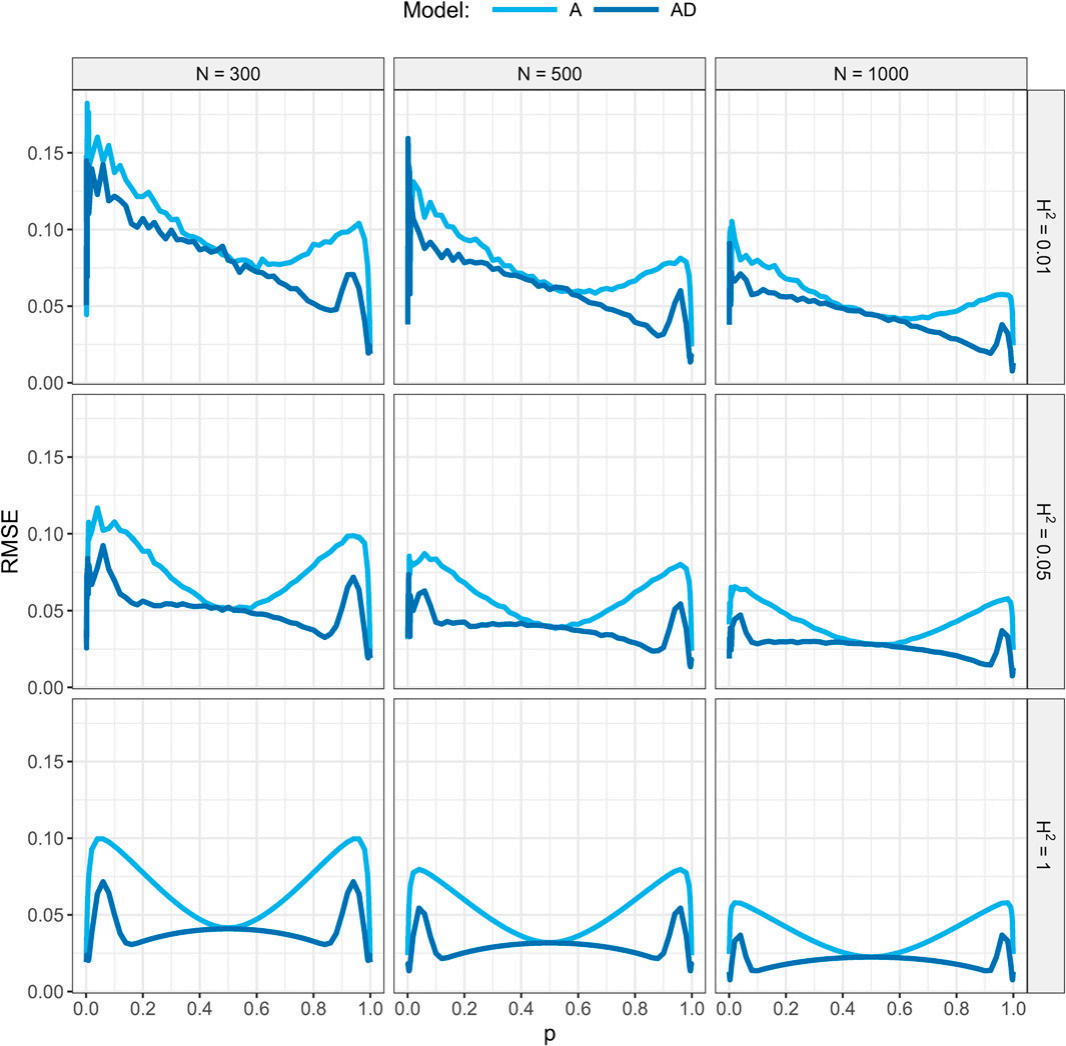
Root mean squared error (RMSE) of $\hat{\alpha }$ with the A- and AD-model. Presented as a function of broad-sense heritability ($H^{2}$), population allele frequency (p), and sample size (N). The additive and dominant effect of the gene were both equal to one.

Figure 1 shows the RMSE of $\hat{\alpha }$ with the A- or AD-model, for a=1 and d=1. For all scenarios, the RMSE of $\hat{\alpha }$ was smaller with the AD-model than with the A-model.

In scenarios where $H^{2}=1,$ RMSE was symmetrical around p=0.5 with both the A- and AD-model. For brevity, we will therefore only describe the pattern for p<0.5. For both models and all N, the RMSE was smallest when p was close to 0, and increased when allele frequency increased. With the A-model, RMSE was largest around p=0.04 and then decreased when p moved toward 0.5. With the AD-model, RMSE was also largest around p=0.04, then decreased when p moved toward 0.1, after which RMSE slightly increased again until p=0.5.

With $H^{2}<1,$ RMSE showed a similar pattern, but was not symmetrical around p=0.5. Compared with $H^{2}=1,$ the RMSE was larger for all p, but this contrast decreased when p increased. This asymmetry was a result of fixing $H^{2}$ in the simulations, which caused the ratio of the dominance variance and residual variance to increase with p. For all scenarios, RMSE decreased when N increased.

**Figure 2:**
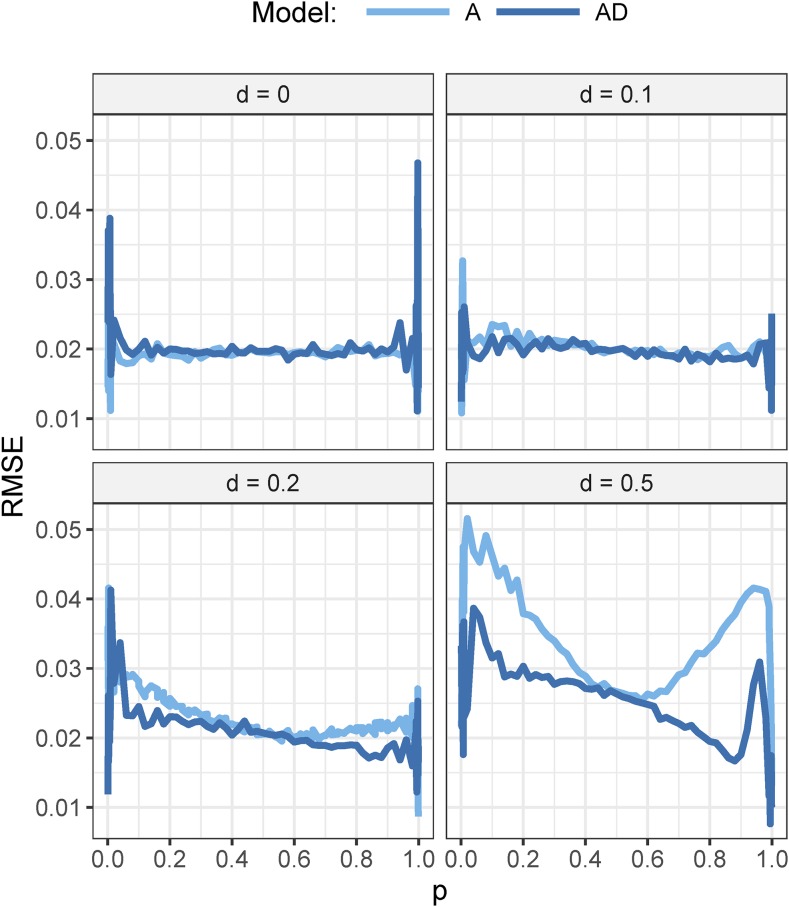
Root mean squared error (RMSE) of $\hat{\alpha }$ with the A- and AD-model for several sizes of dominance effect (*d*). Broad-sense heritability is 0.05 and sample size is 500. Presented as a function of population allele frequency (p). Additive effect of the gene was equal to one.

Figure 2 shows the RMSE of $\hat{\alpha }$ with the A- or AD-model, for a=1, N=500, $H^{2}=0.05,$ and different dominance effects (d). For d=0 and d=0.1, there was almost no difference in RMSE between the A- and AD-model. This indicates that in the absence of dominance, there was no disadvantage of using the AD-model in terms of RMSE. For d=0.1, there was no apparent benefit from using the AD-model. For d=0.2 and d=0.5, however, the AD-model had lower RMSE than the A-model.

### Contribution of finite sampling deviation to the root mean squared error

When there is no environmental variance ($H^{2}=1$) and the model is correct, the RMSE of $\hat{\alpha }$ is expected to be zero. The results, however, show that the RMSE is larger than zero with both the A- and AD-model. To gain more insight into the sources of this error, we investigated the contribution of single samples to the RMSE, for one scenario where $H^{2}=1,$ N=300, p=0.10, and a=d=1, so that α=1.8. For this purpose, we studied the squared difference between $\hat{\alpha }\left(c\right)$ and α (*i.e.*, squared error), as a function of the realized number of individuals with genotype 2 ($n_{2}$). The samples have different probabilities of occurring, so that some samples may contribute more to the total RMSE than others. We therefore investigated the contribution of finite sampling deviation to the RMSE (δ(c)), by weighting the squared errors of $\hat{\alpha }$(c) with their probabilities (see Equation 11).

#### Additive model:

**Figure 3:**
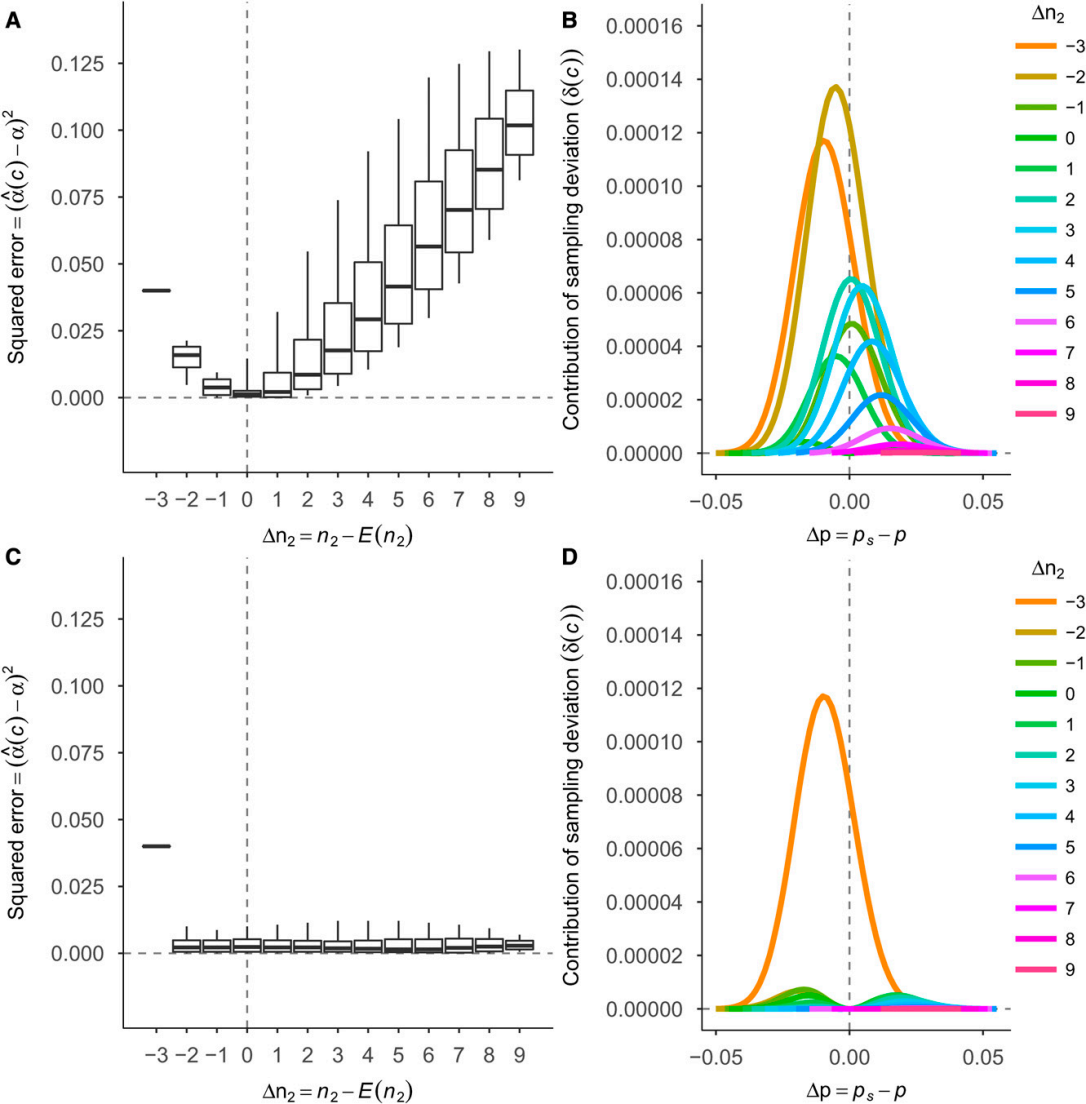
Squared errors of $\hat{\alpha }$ and contributions of samples to the RMSE for the A-model (A and B) and AD-model (C and D), for N=300, $H^{2}=1,$ and p=0.10. The additive and dominant effects of the gene were both equal to one. (A and C) Squared error of $\hat{\alpha }\left(c\right)$ as a function of the departure of $n_{2}$ from its expected value under HWE ($\Delta n_{2}$). (B and D) The effect of $\Delta n_{2},$ and deviations of sample allele frequency from population allele frequency, on the contributions of samples to the RMSE of $\hat{\alpha }.$

Figure 3A shows the squared error as a function of the realized number of individuals with genotype 2, for the A-model. The realized number of individuals with genotype 2 in the sample is expressed as a departure from its expectation (*i.e.*, $\Delta n_{2}$), where the expectation is $E\left(n_{2}\right)=p^{2}N=3.$ The squared error was smallest when $\Delta n_{2}$ was zero and increased as $\Delta n_{2}$ moved away from zero. The remaining variance in squared error for a given value of $\Delta n_{2}$ (as shown by the boxplots) was due to variation in the difference between $p_{s}$ and p (*i.e.*, Δp). For example, when $\Delta n_{2}=3,$ the allele frequency in the sample can vary, because the number of sampled heterozygotes can vary. This variation in Δp affects $\hat{\alpha }\left(c\right),$ except when $\Delta n_{2}=-3.$ In that case, the number of individuals with genotype 2 was zero (in this example) and $\hat{\alpha }\left(c\right)$ was always the slope of a line between two data points.

Figure 3B shows the effect of $\Delta n_{2}$ and Δp on δ(c) for the A-model. The sample where $\Delta n_{2}=0$ and Δp=0 did not contribute to the RMSE (δ(c)=0). Samples where $\Delta n_{2}<0$ had the largest contributions to the RMSE, and samples where $\Delta n_{2}>0$ had somewhat smaller contributions. Figure 3B also shows that Δp contributed less to the RMSE than $\Delta n_{2},$ because there were samples where Δp=0, but δ(c) was relatively large.

#### Additive plus dominance model:

Figure 3C shows the squared error as a function of $\Delta n_{2},$ for the AD-model. The squared error was small and about equal for all $\Delta n_{2},$ except for $\Delta n_{2}=-3,$ where the squared error was largest and exactly the same as with the A-model (see Figure 3A), because there were no individuals with genotype 2 in the sample. Similar to the A-model, the remaining variance (as shown by the boxplots) was due to variation in the difference between $p_{s}$ and p (Δp).

Figure 3D shows the effect of $\Delta n_{2}$ and Δp on δ(c) for the AD-model. Samples where both $\Delta n_{2}\neq -3$ and Δp=0, did not contribute to the RMSE (δ(c)=0). Samples where $\Delta n_{2}=-3$ showed the largest contribution, while all other samples showed small δ(c). Similar to the A-model, Figure 3D shows that Δp was not an important source of error.

#### A- *vs.* AD-model:

In conclusion, even when the locus explains all variance (*i.e.*, $H^{2}=1$), $\hat{\alpha }$ shows error with both the A- and AD-model when it is based on a finite random sample from a population in HWE and dominance is present. With the A-model, the error originated mainly from sampling deviations of genotype frequencies from expected HWE frequencies (ΔHWE), and to a lesser extent from sampling deviations of allele frequencies (Δp) (Figure 3B). With the AD-model, the error originated from Δp only, provided that all three genotype classes were sampled (Figure 3D). These results partly explain the patterns of RMSE in Figure 1 (see Appendix A for more detail).

### Mean RMSE across allele frequency distribution

**Figure 4:**
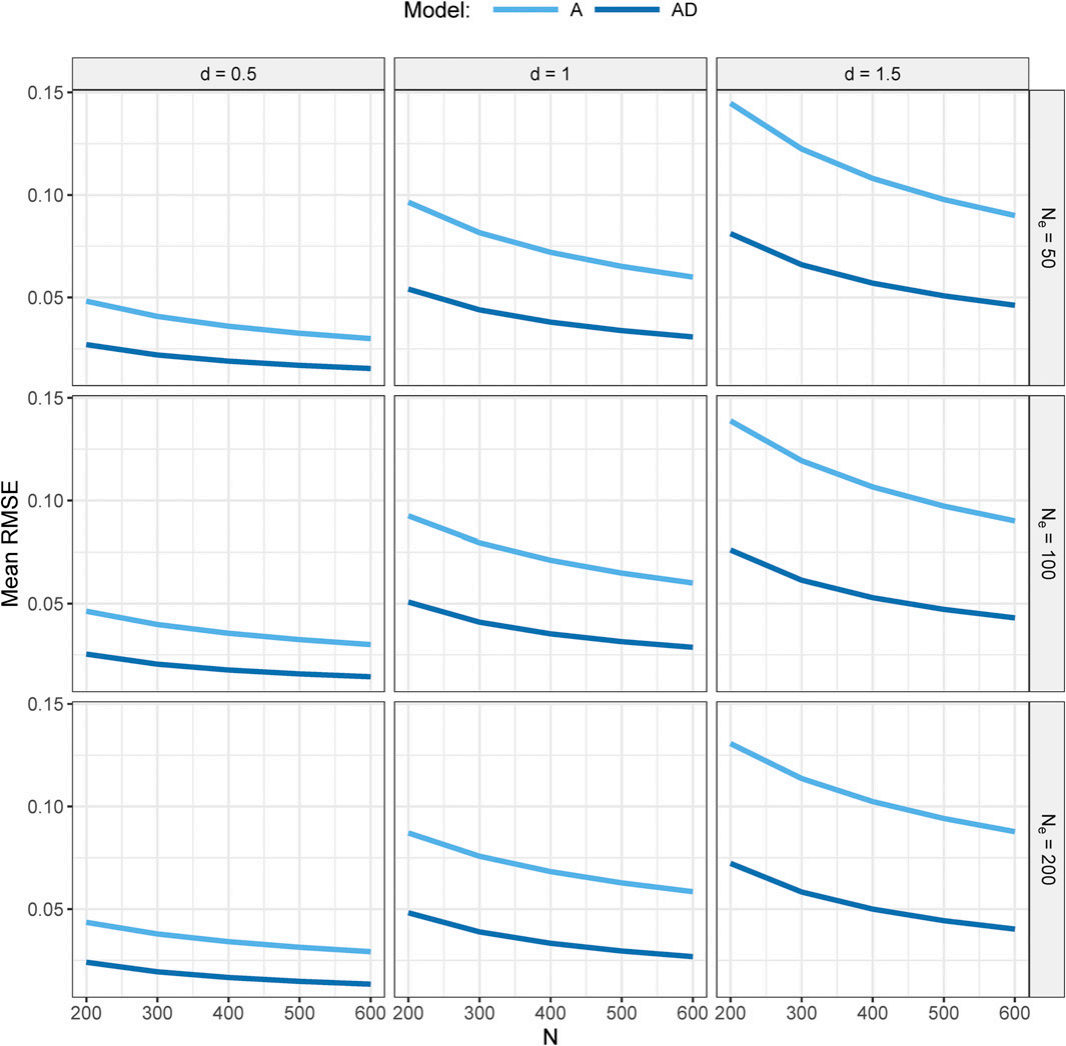
Mean RMSE of $\hat{\alpha }$ for the A- and AD-model, averaged over the distribution of p. Presented as a function of dominance effect (d), sample size (N), and effective population size ($N_{e}$). The distribution of population allele frequencies was assumed to be U-shaped (Equation 13). The broad-sense heritability and the additive gene effect (a) were both equal to one.

Above, we illustrated the RMSE of $\hat{\alpha }$ as a function of p. Now, we present the mean RMSE averaged over the distribution of p, for a=1 and $H^{2}=1,$ assuming a U-shaped distribution of p as a function of $N_{e},$ and for different values for N and d (Figure 4). For all scenarios, the mean RMSE with the A-model was about twice as large as the mean RMSE with the AD-model.

With both models, the mean RMSE was zero when d was zero (data not shown) and increased as d increased. The mean RMSE decreased when N increased. The mean RMSE decreased a little when $N_{e}$ increased, which was caused by differences in the U-shaped distribution of allele frequencies. For example, when $N_{e}=50,$ the percentage of loci with an allele frequency outside the 0.05–0.95 range was 36%, whereas when $N_{e}=200,$ this percentage was 51%. Loci in this range have a low RMSE (see Figure 1 and Appendix A), and therefore a higher $N_{e}$ results in a lower mean RMSE. The effect of $N_{e}$ on the mean RMSE decreased as N increased. Results were identical when a was changed, because the mean RMSE scales linearly with the absolute dominance effect d, and not with the dominance coefficient d/a.

## Discussion

We investigated the accuracy (in terms of RMSE) of estimated average effects ($\hat{\alpha }$) in the presence of dominance, using a single locus model including only an additive or an additive plus dominance effect. In the presence of dominance, the A-model falsely assumes that residuals are IID. The AD-model was therefore expected to better fit the data and give more accurate estimates of α, but only when dominance is present and sample size sufficient for the dominance effect to be accurately estimated. Our results, however, show that the AD-model was always equally or more accurate than the A-model, even with small sample sizes (*i.e.*, N=300), a heritability lower than one (*i.e.*, $H^{2}<1$), or in the absence of dominance.

With the A-model, both sampling deviations of genotype frequencies from HWE frequencies (ΔHWE) and sampling deviations of allele frequencies (Δp) contributed to the error. With the AD-model, only sampling deviations of allele frequencies contributed to the error, provided that all three genotype classes were sampled. The contribution of Δp to the error was much smaller than the contribution of ΔHWE. The AD-model was therefore more accurate than the A-model. Thus, even when the locus explained all variance (*i.e.*, H^2^ = 1), the mean RMSE decreased as sample size increased, because with larger sample sizes, deviations from HWE that considerably affect $\hat{\alpha }$ had a lower probability of occurring. Additionally, with larger sample sizes, the chance of missing one of the genotype classes was smaller, which further reduced the RMSE. The (mean) RMSE of $\hat{\alpha }$ was always smaller with the AD-model than with the A-model. The RMSE of $\hat{\alpha }$ scaled linearly with d; if d doubled, the RMSE also doubled. Remarkably, in the absence of dominance, there was no disadvantage of using the AD-model. Hence, the AD-model yielded equally or more accurate estimates of average effects than the A-model for all scenarios considered.

With the A-model, $\hat{\alpha }$ is computed as the linear regression coefficient of genotypic values on allele counts (Fisher 1941), which yields the average effect in the sample ($\alpha_{s}$), rather than the average effect in the whole population (α). Hence, the expectation of ${\hat{\alpha }}_{A}$ is equal to

$$E\left({\hat{\alpha }}_{A}\right)=\alpha_{s}=a+\left(1-2p_{s}\right)d\left(\frac{1-F_{s}}{1+F_{s}}\right),$$ (14)

where $F_{s}$ measures the deviation from HWE in the sample (ΔHWE) (Haldane 1954; Falconer 1985). Here, $F_{s}$ is defined as one minus the ratio between the observed number of heterozygotes and the expected number of heterozygotes based on the sample allele frequency (Haldane 1954; Wright 1969). With the AD-model, $\hat{\alpha }$ is computed from $\hat{a}$ and $\hat{d},$ which are simultaneously estimated from the data. Unlike the A-model (where $E\left(\hat{\alpha }\right)$ = $\alpha_{s}$), the expectation of ${\hat{\alpha }}_{AD}$ is equal to

$$E\left({\hat{\alpha }}_{AD}\right)=a+\left(1-2p_{s}\right)d,$$ (15)

when all three genotype classes are sampled. Comparison of Equation 14 and Equation 15 shows that the error in ${\hat{\alpha }}_{A}$ originates from both ΔHWE and Δp, while the error in ${\hat{\alpha }}_{AD}$ originates from Δp only, except when one of the genotypes is missing in the sample. When only two genotype classes are sampled, the AD-model reduces to the A-model. With the AD-model, the contrast between the mean genotypic value of the homozygotes and the genotypic value of the heterozygotes (d) does not depend on the number of individuals in these two groups. This is why the AD-model is more robust against deviations from HWE than the A-model.

These results were confirmed by mathematical derivations of the error with the two models (Appendix B). In theory, the error from the A-model can be quantified when $p_{s},$ p, $F_{s}$, and d are known, and from the AD-model when $p_{s},$ p, and d are known. In real data, however, p (and also d with the A-model) is not known, and therefore the error cannot be quantified. As a result, the error cannot be removed from either of the two models. In conclusion, the AD-model is preferred for the estimation of average effects when dominance is present, because it yields more accurate estimates than the A-model, particularly when sample sizes are small.

In this study, we used the so-called genotypic parameterization of the AD-model, as opposed to the breeding parameterization (Vitezica *et al.* 2013). The results, however, were identical to the breeding parameterization (results not shown), because the two parameterizations are equivalent.

Additional to the contribution of dominance to additive variance, evidence for the contribution of epistasis is increasing (Mackay 2015; Monnahan and Kelly 2015). Our results show that modeling dominance improves estimated average effects, and it may therefore be tempting to hypothesize that modeling epistasis may also improve estimates. However, investigating the benefit of modeling epistasis for the accuracy of $\hat{\alpha }$ is not straightforward, because it requires extension to multiple loci.

Taking a finite sample from a large population, which was done in this study, closely resembles a sharp reduction to a small population size, known as a bottleneck. In a small population, genotype frequencies deviate from HWE even under random mating. The expected genotype frequency for heterozygotes is equal to $2p_{s}\left(1-p_{s}\right)\left(1-F_{s}\right)$ (Haldane 1954). In turn, the expectation of $F_{s}$ depends on the size of the bottleneck (or sample size,N), and is equal to −1/(2N−1) with random mating (Kimura and Crow 1963). This indicates that the expected heterozygosity in the sample is larger than the HWE frequency calculated from the sample allele frequency. The effect of ΔHWE on estimated average effects was studied by Wang *et al.* (1998), who focused on the consequences for the additive genetic variance. In agreement with our results, they showed that the average effect was not influenced by ΔHWE when d=0, or when p≈0.5. Furthermore, the effect of ΔHWE on estimated average effects depended on the size of the bottleneck (or sample size, N) and the size of dominance effect (d) (Wang *et al.* 1998). Because the effects of a bottleneck are very similar to the effects of taking a small sample from a large population, the results of our study also apply to populations in a bottleneck.

We quantified the error in estimates of α that originated from ΔHWE in random finite samples from a population of unrelated individuals. We purposefully used relatively small sample sizes to illustrate the effect. Although sample sizes taken in empirical studies may be larger, effective sample size may be much smaller, because actual populations often have small effective population size ($N_{e}$) (Hall 2016). This low $N_{e}$ is related to the family structure in the population, where many individuals are bred from a limited number of parents, so that $N_{e}$≪ N. Hence, the effective sample size may be much smaller than N, because the sample will partly consist of related individuals. Because of this relatedness, sampling deviations in allele and genotype frequencies can be larger than expected based on sample size. The sample sizes chosen in this study may therefore be similar to effective sample sizes in empirical studies. As an example, we investigated the SD of $F_{s}$ across allele frequencies in a dataset of ∼3500 pigs (Cleveland *et al.* 2012). The resulting value was comparable to the expected SD of $F_{s}$ for samples of 500–1000 animals (see Appendix C), which supports our expectation that effective number of sampled individuals may be smaller than the actual number of sampled individuals. Furthermore, in many studies that use genotype data, markers are removed if they show a significant deviation from HWE. The significance threshold that is used for HWE filtering, however, is often very liberal (Gondro *et al.* 2013). Consequently, there are still many markers left in the data that deviate from HWE and may give inaccurate estimates of average effects. As a result, we expect that the magnitude of ΔHWE simulated in this study may be similar to ΔHWE in empirical studies.

The estimation of average effects at single loci, as presented in this study, may be relevant for genome-wide association studies (GWAS). In GWAS, a large number of markers spread across the genome are each tested for an association with the observed phenotype (Gondro *et al.* 2013). Most GWAS test these associations by using an additive model which treats the marker genotypes as fixed (Hayes 2013). Only few studies have used the AD-model in GWAS to explicitly estimate a and d (*e.g.*, Lopes *et al.* 2014; Aliloo *et al.* 2015; Huang *et al.* 2015; Bennewitz *et al.* 2017) and, to our knowledge, none have investigated differences in accuracy of estimated average effects between the A-model and AD-model. The effects of sampling genotypes on $\hat{\alpha }$ shown in this study apply to ${\hat{\alpha }}_{m}$ in GWAS, because ${\hat{\alpha }}_{m}$ are usually estimated by ordinary least squares. Using the AD-model in GWAS will therefore yield more accurate estimates of average effects and explained variance of markers.

The results presented in this study may also be relevant for genomic prediction. In genomic prediction, genomic estimated breeding values (GEBVs) are calculated as the sum of many estimated average effects multiplied by their marker genotypes (Meuwissen *et al.* 2001). Differences in accuracy of GEBVs may therefore be related to differences in accuracy of the estimated average effects. Our results, however, cannot be extrapolated directly to accuracy of GEBVs for several reasons. In this study, we considered a single locus, estimated α as a fixed effect, and assumed known genotypes of the quantitative trait locus (QTL). By contrast, GEBVs are based on many marker loci, for which all α’s are estimated simultaneously as random effects (Meuwissen *et al.* 2001). In genomic prediction, the effect of a single QTL is likely to be explained by multiple markers, and errors of individual marker effects may cancel out to some extent when accumulated within individuals to compute their GEBVs. Additionally, random effect models shrink average effects toward zero (Whittaker *et al.* 2000), which may shrink the sampling error as well. In conclusion, to translate our results to accuracy of GEBVs, this research should be extended to the estimation of multiple random effects based on marker genotypes.

Neither GWAS nor genomic prediction are based on the genotypes at QTL directly, but rely on linkage disequilibrium (LD, measured by r) between observed markers and unknown QTL (Lewontin and Kojima 1960). For the additive effect at the QTL, the fraction captured by the marker is proportional to r, whereas for the dominance effect, the fraction captured by the marker is proportional to $r^{2}$ (Weir 2008; Zhu *et al.* 2015). The proportion of the signal of the dominance part of $\alpha_{m}$ that is captured is therefore expected to be smaller than of the additive part, because $r^{2}\leq r.$ For this reason, a marker should be very close to a QTL to pick up its dominance effect (Wellmann and Bennewitz 2012). As a result, the benefit of dominance models over additive models may be smaller with lower marker densities. We therefore argue that, when dominance is present and markers are able to capture dominance, the dominance model yields more accurate estimates of α than the additive model.

## Conclusions

When a single locus average effect is estimated in a random finite sample from a large population in HWE, both A-models and AD-models yield error in their estimates, even when the locus explains all variance (*i.e.*, $H^{2}=1$). Estimates from the AD-model, however, are more robust against chance deviations from HWE frequencies than estimates from the A-model. Genetic models that include dominance, therefore, yield higher accuracies of estimated average effects at single loci than purely additive models when dominance is present. In the absence of dominance, there was no penalty for fitting dominance. These results are important for GWAS, and potentially also for genomic prediction.

## Appendix A

With the A-model, error originated mainly from sampling deviations of genotype frequencies from expected HWE frequencies (ΔHWE), and to a lesser extent from sampling deviations of allele frequencies (Δp). For all N, the RMSE was smallest at intermediate allele frequencies, and increased when allele frequency moved away from 0.5 (Figure 1). This increase was due to the increasing probability of sampling fewer individuals from the rare genotype class than expected based on HWE. The RMSE decreased again at extreme allele frequencies. This decrease was due to (1) the increasing probability of not sampling the rare homozygous genotype, and (2) the change in α. When one of the homozygous genotypes is not sampled, $\hat{\alpha }$ is always equal to the slope of the regression line between the opposing homozygotes and the heterozygotes (a+d when $n_{2}=0,$ or a−d when $n_{0}=0$). As the allele frequency approaches fixation, the probability of missing a homozygous genotype increases, while at the same time, the true α becomes close to the slope of the resulting regression line (see Equation 1) (a+d when p→0, or a−d when p→1). Hence, the RMSE as a result of missing one genotype class was small at extreme allele frequency and increased as the allele frequency increased. It is good to note that with only one genotype class sampled, the sample was disregarded because $\hat{\alpha }$ could not be estimated.

With the AD-model, error originated from Δp only, provided that all three genotype classes were sampled. For all N, the RMSE with the AD-model was always smaller than with the A-model, and showed a different pattern across allele frequencies (Figure 1). The RMSE decreased slightly when allele frequency moved away from 0.5, because the probability of drawing a sample with a very high Δp decreased. RMSE increased again for allele frequencies ∼0.1 or 0.9. This increase was due to the increased probability of sampling no individuals from the rare genotype class, in which case the AD-model reduced to the A-model. The RMSE decreased again at even more extreme allele frequencies, for the same reasons as explained for the A-model.

## Appendix B

Our aim is to estimate α in the entire population, but with the A-model, the estimate we get from the sample is equal to $\alpha_{s}.$ So, we can predict what the error will be by deriving the difference between $\alpha_{s}$ and α.

$$error=E\left({\hat{\alpha }}_{A}\right)-\alpha =\alpha_{s}-\alpha =\left[a+\left(1-2p_{s}\right)d\left(\frac{1-F_{s}}{1+F_{s}}\right)\right]-\left[a+\left(1-2p\right)d\right]=\left(1-2p_{s}\right)d\left(\frac{1-F_{s}}{1+F_{s}}\right)-\left(1-2p\right)d=\left(\frac{1-F_{s}}{1+F_{s}}\left(1-2p_{s}\right)-\left(1-2p\right)\right)d$$

Then, if there is no deviation from HWE (F=0), the error due to deviations of $p_{s}$ from p is

$$\left[\left(1-2p_{s}\right)-\left(1-2p\right)\right]d=2\left(p-p_{s}\right)d.$$

Similarly, if $p_{s}=p,$ the error due to deviations from HWE is equal to

$$\left(\frac{1-F_{s}}{1+F_{s}}-1\right)\left(1-2p_{s}\right)d.$$

When we correct for deviations from HWE, assuming that we know d, we get

$$\begin{array}{c} {\hat{\alpha }}_{c}=\alpha_{s}-error=\left[a+\left(1-2p_{s}\right)d\left(\frac{1-F_{s}}{1+F_{s}}\right)\right]-\left[\left(\frac{1-F_{s}}{1+F_{s}}-1\right)\left(1-2p_{s}\right)d\right]\\ =a+\left(1-2p_{s}\right)d\left(\frac{1-F_{s}}{1+F_{s}}\right)-\left(1-2p_{s}\right)d\left(\frac{1-F_{s}}{1+F_{s}}\right)+\left(1-2p_{s}\right)d\\ =a+\left(1-2p_{s}\right)d. \end{array}$$

If we subsequently correct for deviations of p, assuming that we know p, we get

$$\left[a+\left(1-2p_{s}\right)d\right]-\left[2\left(p-p_{s}\right)d\right]=\left[a+\left(1-2p_{s}\right)d\right]-\left[\left(1-2p_{s}\right)-\left(1-2p\right)\right]d=a+\left(1-2p\right)d,$$

which is the average effect in the population.

For the AD-model, we can predict error for samples that have all three genotype classes by

$$\begin{array}{c} error=E\left({\hat{\alpha }}_{AD}\right)-\alpha =\left[a+\left(1-2p_{s}\right)d\right]-\left[a+\left(1-2p\right)d\right]\\ =\left[\left(1-2p_{s}\right)-\left(1-2p\right)\right]d\\ =2\left(p-p_{s}\right)d, \end{array}$$

which is equal to the error due to deviations of $p_{s}$ from p with the A-model.

## Appendix C

Appendix C Expected SD of *F_s_* for several sample sizes, compared with the SD of *F_s_* measured in an empirical dataset of pigs.
